# Post-cesarean Delivery Analgesia Using Spinal Anesthesia: Ropivacaine-Fentanyl vs. Ropivacaine-Sufentanil

**DOI:** 10.5812/aapm-138067

**Published:** 2023-07-26

**Authors:** Ahmad Reza Mohtadi, Atusa Ahmadi Chegeni, Kaveh Behaeen, Mohsen Savaie, Ali Ghomeishi

**Affiliations:** 1 Department of Anesthesiology, School of Medicine, Ahvaz Jundishapur University of Medical Sciences, Ahvaz, Iran; 2 Pain Research Center, Ahvaz Jundishapur University of Medical Sciences, Ahvaz, Iran

**Keywords:** Spinal Anesthesia, Fentanyl, Sufentanil, Ropivacaine, Analgesia, Cesarean Section

## Abstract

**Background:**

To improve the quality of intraoperative and postoperative analgesia during spinal anesthesia, intrathecal opioids are used as adjuvant drugs in combination with local anesthetics.

**Objectives:**

This study aimed to compare the intrathecal injection of ropivacaine-fentanyl with ropivacaine-sufentanil in terms of the duration of analgesia after cesarean section (CS).

**Methods:**

This randomized, double-blind clinical trial study was conducted on women referred to Imam Khomeini Hospital of Ahvaz City for elective CS in 2021. A total of 51 patients were randomly divided into 2 groups. The first group (n = 25) received ropivacaine (17.5 mg) + fentanyl (25 μg), while the second group (n = 26) received ropivacaine (17.5 mg) + sufentanil (2.5 μg) for spinal anesthesia. Eventually, several parameters were investigated, including the duration of sensory and motor block, duration of analgesia (based on the Visual Analog Scale (VAS)), hemodynamic parameters, and possible complications.

**Results:**

The duration of surgery (P = 0.059) and the duration of motor block (P = 0.962) were not significantly different between the 2 groups. The mean duration of analgesia (from the time of entering recovery to reaching VAS = 3) was 203.12 ± 72.93 and 207.46 ± 69.59 minutes in the fentanyl and sufentanil groups, respectively (P = 0.658). Systolic and diastolic blood pressure (SBP/DBP) drops in minute 5 were observed more frequently in the sufentanil group than in the fentanyl group (P = 0.027 and P = 0.002, respectively). At the other time points, however, no significant difference was observed between the 2 groups in terms of hemodynamic variables (P > 0.05). Finally, the frequency of pruritus was higher in the sufentanil group than in the fentanyl group (26.9% vs. 4.0%; P = 0.024).

**Conclusions:**

Adding fentanyl or sufentanil to intrathecal ropivacaine provides a similar duration of analgesia. However, fentanyl was associated with better hemodynamic stability and a lower incidence of pruritus.

## 1. Background

Cesarean section (CS) is one of the most common surgical procedures for women in both developed and developing countries (1). The rates of CS are alarmingly on the rise worldwide (2), and according to the World Health Organization (WHO), 21% of newborns are born through CS (3). In Iran, this rate has been reported to be 53.6% (4). The method of anesthesia and analgesia plays a pivotal role in the quality of treatment and care of these patients, and the most common method used for anesthesia in CS is spinal anesthesia, which is employed in 90% and 80% of elective and emergency CSs, respectively (5).

Ropivacaine is a common local anesthetic in spinal anesthesia (6). Compared to bupivacaine, ropivacaine is associated with fewer cardiac complications, shorter motor blocks, and faster recovery. The potency of ropivacaine is equivalent to 0.6 of bupivacaine (7, 8). Combining opioids with ropivacaine has been shown to have a synergistic effect on spinal anesthesia, resulting in a faster onset of sensory and motor block, a lower dose of ropivacaine required, fewer complications related to the dosage of both drugs, fewer hemodynamic changes, and longer and improved quality of postoperative analgesia (7-9).

Lipophilic opioid drugs are usually used as adjuvant drugs in combination with local anesthetics to reduce the dose and, thus, the side effects of local anesthetics in CS (10, 11). Lipophilic opioids (such as fentanyl and sufentanil) have better spinal effects than hydrophilic opioids. Fentanyl and sufentanil have a faster onset of action, more rapid motor recovery, and less upward spread, reducing the risk of respiratory depression (12). Sufentanil is twice as fat-soluble as fentanyl. The effect of 2 - 10 μg of sufentanil is comparable to 25 μg of fentanyl (13). Intrathecal fentanyl is also 10 - 20 times more effective than intravenous fentanyl (10). Although fentanyl and sufentanil have been administered through intrathecal injection for years, there are no definite recommendations regarding the use of these drugs; the main concern about their use is the occurrence of respiratory depression (14). A meta-analysis of women undergoing spinal anesthesia with different doses of fentanyl reported no respiratory depression (10). Despite the high rate of CS and the importance of postoperative pain control, few studies with different study methods (various drug combinations and doses of drugs) have been conducted, yielding conflicting results regarding the effectiveness of spinal fentanyl and sufentanil analgesia in CS (6, 14). Therefore, it is necessary to identify the best drug choice that can control the pain after CS with a simple and economical method.

## 2. Objectives

The present study was thus conducted to compare the intrathecal injection of ropivacaine-fentanyl with ropivacaine-sufentanil in terms of the duration of analgesia after CS under spinal anesthesia.

## 3. Methods

After obtaining approval from the Ethics Committee of Ahvaz Jundishapur University of Medical Sciences (code: IR.AJUMS.HGOLESTAN.REC.1400.084), this randomized, double-blind clinical trial was performed on patients undergoing elective cesarean surgery in the operating room of Imam Khomeini Teaching Hospital of Ahvaz in 2021. The study was also registered on the Iranian Registry of Clinical Trials website (code: IRCT20210827052297N1). Written informed consent was obtained from all patients before starting the treatment. In addition, the provisions of the ethics statement in health research and the principles of confidentiality of patient information were strictly observed in all the stages of this research.

Based on a similar article (6) and considering an alpha error of 0.05 and a power of 90%, the sample size was calculated to be 21 in each group using the following formula:


$$n=\frac{{\left(Z_{1-\frac{\alpha }{2}}+Z_{1-\;\beta }\right)}^{2}(S_{1}^{2}+S_{2}^{2})}{{\left({\overset{-}{X}}_{1}-\;{\overset{-}{X}}_{2}\right)}^{2}}$$

Where:


$$Z_{1-\frac{\alpha }{2}}=1.96$$

$$Z_{1-\;\beta }=1.28$$

$$S_{1}=32.05$$

$$S_{2}=45.05$$

$${\overset{-}{X}}_{1}-\;{\overset{-}{X}}_{2}=40$$

**Figure 1.**
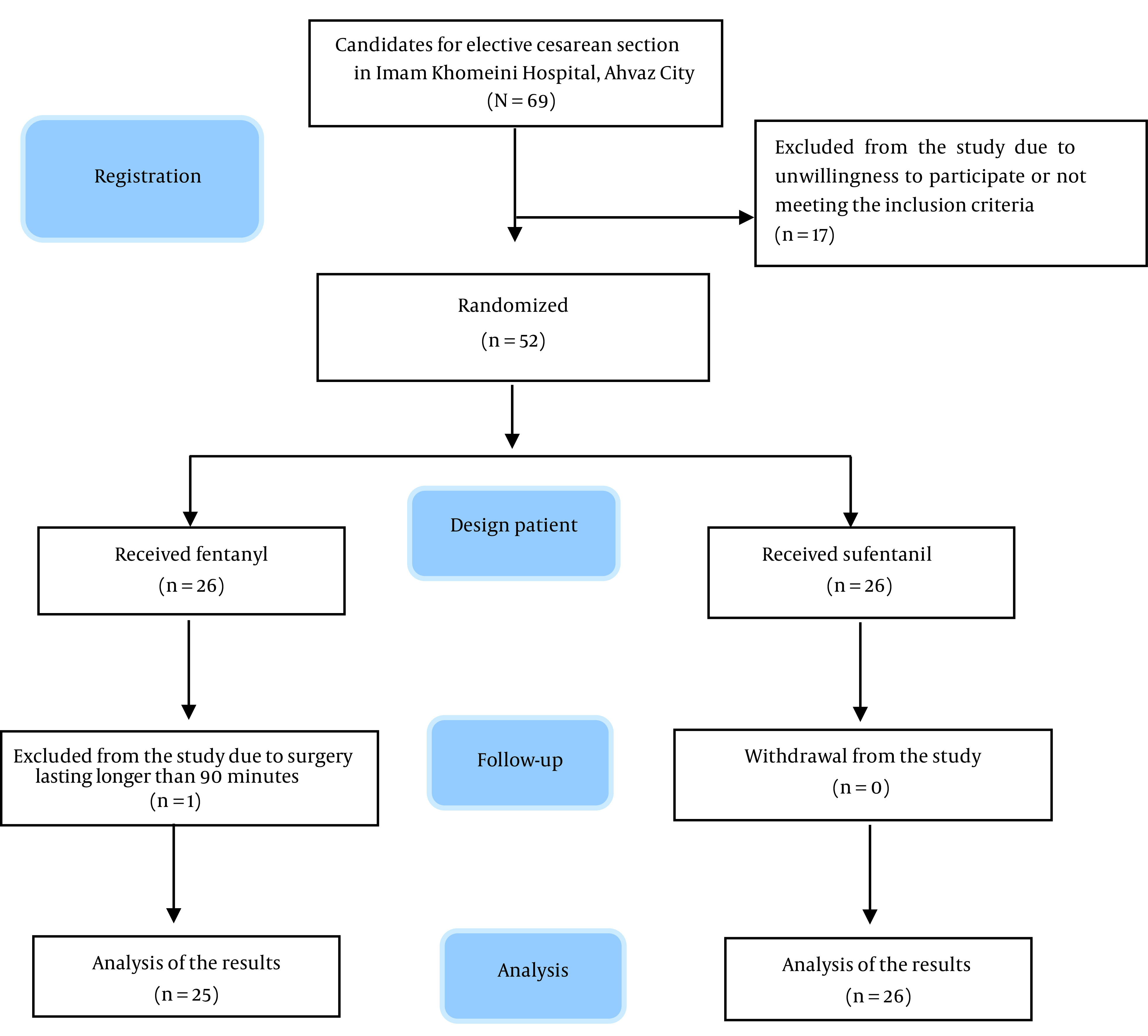
Study flowchart

Considering a 10% attrition rate, the number of studied samples was set as 24 people in each group. The participants were selected using the purposeful sampling method; this method, which is also known as judgmental, selective, or subjective sampling, is a form of non-probability sampling in which researchers rely on their own judgment when choosing the members of the population to participate in their surveys. Inclusion criteria were elective CS patients aged 17 - 45 years with American Society of Anesthesiologists (ASA) classes I and II, no history of addiction, no contraindications to spinal anesthesia, and no allergy to opioids or local anesthetics. Exclusion criteria were simultaneous surgery, surgery lasting longer than 90 minutes, a volume of bleeding more than 1500 mL during surgery, low sensory level for cesarean surgery, or need for general anesthesia. The diagram of the study process (Consolidated Standards of Reporting Trials (CONSORT)) and the exclusion of participants is shown in Figure 1.

### 3.1. Group Allocation and Intervention

At the beginning of the study, the basic characteristics of women, including age, height, weight, and body mass index (BMI), were collected and recorded before their entry into the operating room. Standard monitoring, including electrocardiography, pulse oximetry, and blood pressure (BP) measurement, was performed after participants entered the operating room. For each patient, an 18-gauge angiocatheter was implanted in both hands, and normal saline (10 mL/kg) was infused within 15 to 30 minutes.

The participants were randomly assigned to fentanyl and sufentanil groups using the random block method based on the first random permutation of 4. Randomization was performed by a person who was not involved in the study process.

Spinal anesthesia was administered in the sitting position using a Quinke needle (No. 25, Dr. Japan Company, Japan) in the L3 - L4 or L4 - L5 space based on the Medline approach, which involved the intrathecal injection of ropivacaine with fentanyl or with sufentanil. The sensory and motor block was confirmed by the pinprick test and leg movement.

In the fentanyl group, 3.5 mL of isobaric ropivacaine (17.5 mg; hydrochloride Molteni, 5 mg/mL) and 25 μg of fentanyl (Aburaihan Pharmaceutical Company, Iran) were injected intrathecally. In the sufentanil group, 3.5 mL of ropivacaine (17.5 mg) and 2.5 μg of sufentanil (Aburaihan Pharmaceutical Company, Iran) were injected intrathecally. In both groups, the intrathecal injection volume was 4 mL, and the volume of each fentanyl and sufentanil was 0.5 mL. A combination of ropivacaine and fentanyl or sufentanil was injected intrathecally according to the national guidelines (based on the protocol of Iran’s Ministry of Health for painless delivery). These drugs were used under sterile conditions.

The syringe containing the drug was prepared in advance, and the liquid and drugs were stored at room temperature. The person responsible for the randomization and allocation of people to groups was blinded to the condition of the patients. Moreover, the patients, data collectors, and data analyzers were also blinded to the type of drug received and the grouping.

### 3.2. Evaluation of Outcomes

The primary outcome was the duration of analgesia, and the secondary outcomes were the duration of sensory and motor block, hemodynamic changes, and side effects.

After spinal anesthesia, the patients were placed in the supine position, and the degree of sensory and motor block was measured. The patients’ sensory block (below the surface of the fourth thoracic vertebra) was evaluated based on the pinprick method by examining the loss of sensation to a 7G needle in the midclavicular line. The degree of motor block was assessed and recorded using the Bromage scale ranging from 0 to 3 (0, no paralysis; 1, only the knee moves; 2, only the sole of the foot moves, and 3, the knee and foot are unable to move).

Systolic and diastolic blood pressure (SBP/DBP) and heart rate (HR) were measured and recorded before spinal anesthesia, after spinal anesthesia, every 5 to 20 minutes, and at minutes 30, 40, and 60. If the patient’s SBP dropped to less than 90 mm Hg, 5 mg of ephedrine was administered intravenously.

Pain intensity was evaluated based on the Visual Analog Scale (VAS), with the patient giving a score from 0 (no pain) to 10 (the most severe pain). Pain intensity was estimated upon entering the recovery room (considered as 0 hours), after 15 minutes and 30 minutes, in the first and second hour, every hour for 12 hours, and then every 2 hours for 24 hours. If the patient reported a pain intensity of 3 according to the VAS, 50 mg of diclofenac suppository was administered, and the time of administration and dose were recorded. The duration of analgesia was considered from the time of administering spinal anesthesia until reaching VAS = 3 and from the time of entering recovery until reaching VAS = 3.

All patients were evaluated in the first 24 hours after surgery to check side effects related to the use of drugs. These included nausea, vomiting, and pruritus that were managed under the supervision of an anesthesiologist if necessary.

### 3.3. Statistical Analysis

SPSS version 22 (SPSS Inc, Chicago, IL, USA) was used to perform statistical analyses. Means, SDs, medians, interquartile ranges (IQRs), frequencies, and percentages were used to describe the data. The normality of the data was checked by the Kolmogorov-Smirnov test. The non-parametric Mann-Whitney test was used to compare the mean variables between the 2 groups, and the chi-square test was used to compare qualitative variables. The repeated measures analysis of variance was also used to compare quantitative variables before and after the intervention (within-group comparison), and the significant level in all tests was set at 0.05.

## 4. Results

Fifty-one pregnant women who were candidates for elective CS under spinal anesthesia were studied in 2 groups. Data related to demographic characteristics, motor block, and duration of analgesia in the patients of the 2 groups are presented in Table 1. Demographic characteristics, including age, height, weight, and BMI, were not significantly different between the 2 groups (P > 0.05). Similarly, the duration of surgery (P = 0.059) and the duration of motor block (P = 0.962) were not significantly different between the 2 groups. Moreover, the duration of analgesia from the time of administering spinal anesthesia to reaching VAS = 3 and from the time of entering the recovery room to reaching VAS = 3 did not significantly differ between the 2 groups (P = 0.658 and P = 0.699, respectively).

**Table 1.** Characteristics of the Participants ^a^

Variables	Fentanyl (n = 25)	Sufentanil (n = 26)	P Value ^b^
**Demographic characteristics**			
**Age (y)**	5.02± 29.16	5.16 ± 27.65	0.223
**Height (cm)**	11.58 ± 160.32	4.10 ± 162.04	0.778
**Weight (kg)**	12.75 ± 83.08	16.60 ± 77.60	0.173
**BMI (kg/m** ^**2**^ **)**	6.22 ± 32.65	6.20 ± 29.83	0.066
**Surgery characteristics**			
**Duration of surgery (min), median (IQR)**	52 (47.0 - 58.0)	61 (49.0 - 78.25)	0.059
**Duration of the motor block from the time of spinal anesthesia (min), median (IQR)**	109 (92 - 127.5)	110 (90 - 118.25)	0.962
**Duration of analgesia from the time of administering spinal anesthesia (min)**	196 (165 - 239.5)	208 (142.5 - 279.25)	0.658
**Duration of analgesia from the time of entering the recovery room (min)**	110 (72.5 - 160)	110 (68.75 - 186.25)	0.699
**VAS score in the recovery room**	0.00 ± 2.00	0.19 ± 2.04	0.327

Abbreviations: BMI, body mass index; VAS, Visual Analog Scale.

^a^ Values are expressed as mean ± SD or median (interquartile range).

^b^ Mann-Whitney test.

Changes in the pain score (VAS) from 0 hours (recovery) to the 240th minute in the 2 groups are depicted in Figure 1. There was no significant difference between the 2 groups in terms of pain intensity (VAS score) in any of the time intervals (entry to recovery, 15, 30, 60, 120, 180, and 240 minutes; P < 0.05). After the 240th minute, the pain intensity of the patients was evaluated every hour for 12 hours and every 2 hours for 24 hours. In both groups, pain intensity was a VAS score of less than 3, and there was no need to administer analgesics. Furthermore, no significant difference was found in pain intensity (VAS score) between the 2 groups (P < 0.05).

**Figure 2.**
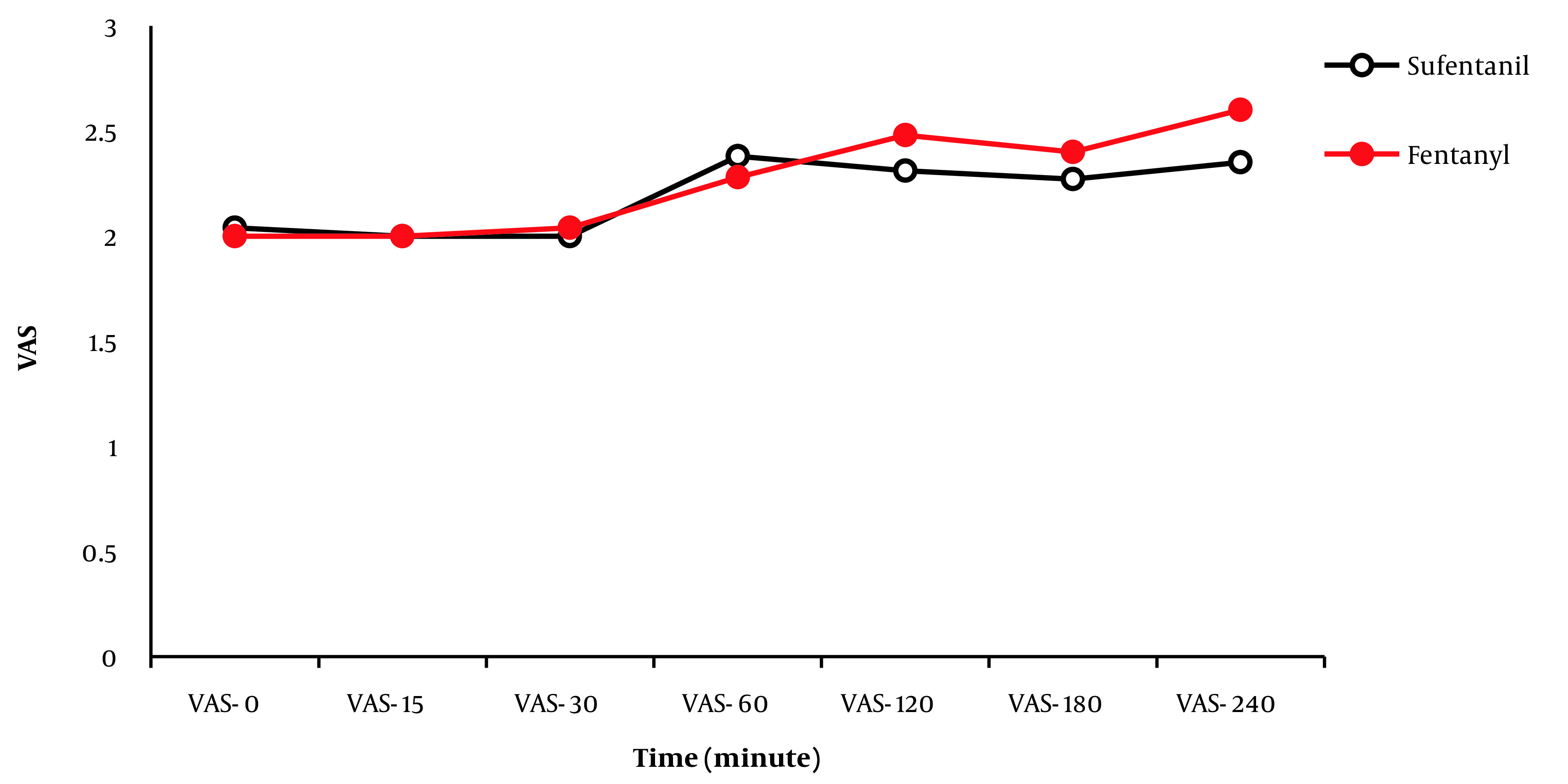
Pain score changes (Visual Analog Scale (VAS)) from 0 hours (recovery) to the 240th minute in the 2 groups

Changes in SBP, DBP, and HR in the 2 groups are displayed in Figure 2. The Mann-Whitney test results indicated that there was no significant change in BP and HR at different time intervals between the 2 groups (P < 0.05). However, only at the fifth minute SBP and DBP were significantly lower in the sufentanil group than in the fentanyl group (P = 0.027 and P = 0.002, respectively).

The Wilcoxon test was used to analyze the changes in BP and HR across different time intervals in both groups. In the sufentanil group, a reduction in SBP and DBP was observed before and after spinal anesthesia (P = 0.002 and P = 0.020, respectively) and at the fifth minute (P = 0.004 and P = 0. 004, respectively). On the other hand, at the 10th minute, there was an increase in SBP and DBP (P = 0.020 and P = 0.042, respectively). At other intervals, no significant difference was found in SBP and DBP. In the fentanyl group, a drop in SBP was detected only at the fifth minute compared to before spinal anesthesia (P = 0.050). At other intervals, there was no significant difference in SBP and DBP.

**Figure 3.**
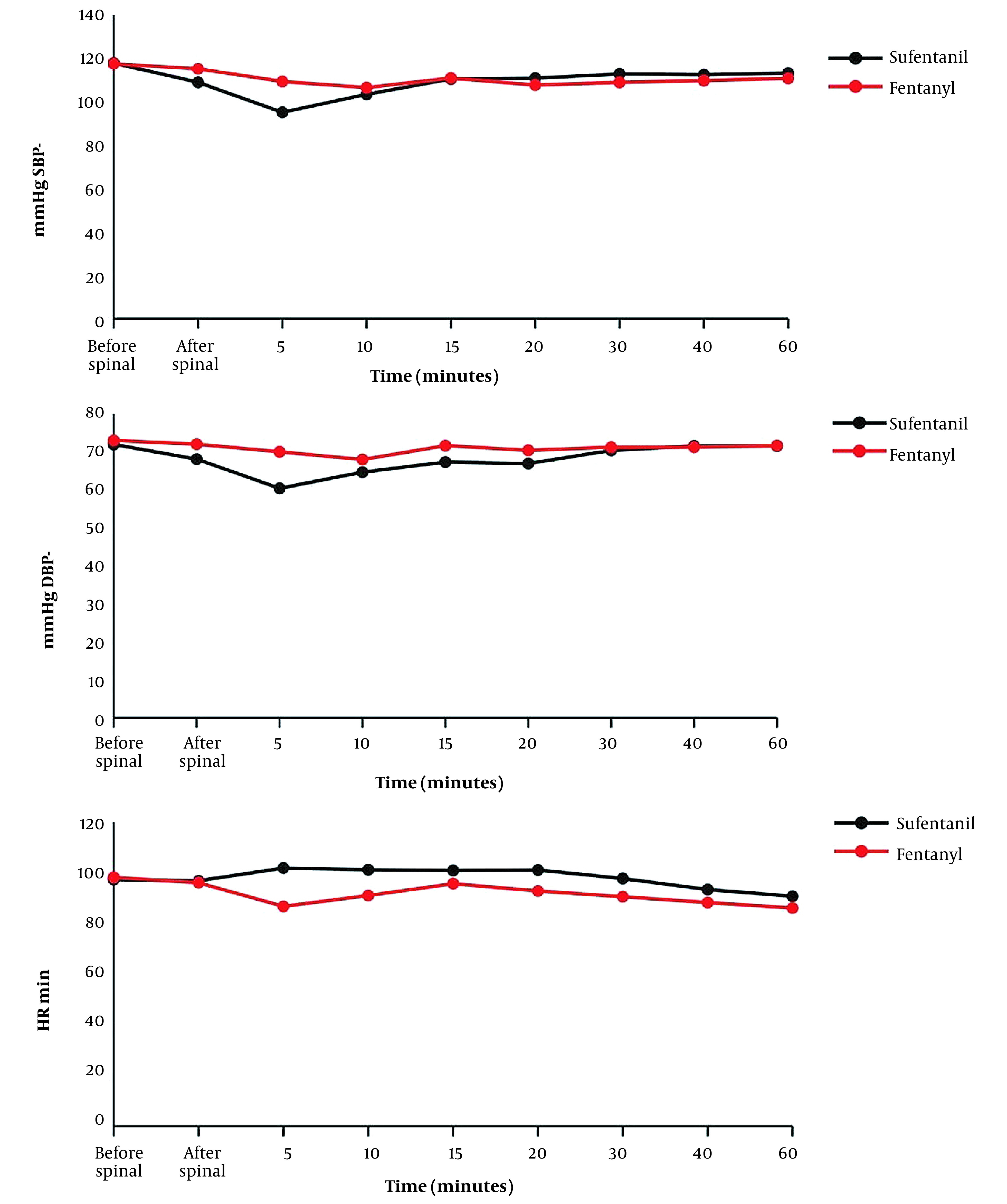
Changes in systolic and diastolic blood pressure (SBP/DBP) and heart rate (HR) in the 2 groups

Based on the within-group comparison, a drop in HR was observed in the fentanyl group at the fifth minute (P = 0.013). Conversely, an increase in HR was found in both sufentanil (P = 0.037) and fentanyl (P = 0.011) groups at the 60th minute. However, no significant changes in HR were detected at other intervals (Figure 3).

Table 2 provides data on the comparison of the need for ephedrine and atropine in the 2 groups. In this study, 12 (46.2%) women in the sufentanil group needed ephedrine 8.42 ± 4.96 minutes after spinal anesthesia, and 7 (26.9%) women required atropine after 7.14 ± 3.93 minutes. Additionally, in the fentanyl group, 7 (28.0%) women needed ephedrine 8.86 ± 4.63 minutes after spinal anesthesia, and 7 (28.0%) women required atropine after 7.00 ± 3.74 minutes. There was no significant difference between the 2 groups in terms of the frequency of needing ephedrine and atropine, as well as the time needed for these 2 drugs.

**Table 2.** Comparison of the Need for Atropine and Ephedrine ^a^

Variables	Fentanyl (n = 25)	Sufentanil (n = 26)	P Value
**Need for atropine**	7 (28)	7 (26.9)	0.931
**Time of need**	3.74 ± 7 (2 - 12)	3.93 ± 7.14 (5 - 15)	0.805
**Need for ephedrine**	7 (28)	12 (46.2)	0.180
**Time of need**	4.63 ± 8.86 (2 - 15)	4.96 ± 8.42 (5 - 20)	0.592

^a^ Values are expressed as No. (%) or mean ± SD (min - max).

In total, 7 (26.9%) women in the sufentanil group and 3 (12%) women in the fentanyl group experienced side effects (nausea, vomiting, and itching; P = 0.180). Table 3 presents data comparing the incidence of vomiting and pruritus between the 2 groups.

**Table 3.** Comparison of Pruritus ^a^

Variables	Fentanyl (n = 25)	Sufentanil (n = 26)	P Value
**Pruritus**	1 (4)	7 (29.6)	0.024
**Vomiting**	2 (8)	0 (0)	0.141

^a^ Values are expressed as No. (%).

## 5. Discussion

The 2 groups did not significantly differ in terms of demographic characteristics (such as age, height, weight, and BMI), indicating that these factors had no influence on the results, and the participants were randomly selected without any bias in sample selection. There was no significant difference between the 2 groups regarding the duration of surgery, the duration of motor block, the duration of analgesia, and changes in pain score (VAS). These results are in line with the findings of previous studies reporting increased quality of analgesia after adding lipophilic opioids, such as spinal fentanyl and sufentanil, to local anesthetics (9, 10, 15). In a meta-analysis study, Fonseca et al. revealed that adding fentanyl and spinal sufentanil to the local anesthetic significantly reduced both postoperative pain and opioid consumption while increasing the duration of analgesia and prolonging the time to administer the first postoperative analgesic agent (14). Farzi et al. evaluated the effect of adding fentanyl, sufentanil, and placebo to intrathecal bupivacaine in patients undergoing CS with spinal anesthesia (6). According to their results, the duration of analgesia (from the end of intrathecal injection to reaching a VAS score of 4) in the fentanyl and sufentanil groups was 314 and 312.5 minutes, respectively, which was significantly longer than that in the placebo group (116.1 minutes). Furthermore, the duration of sensory and motor block was longer in the fentanyl and sufentanil groups compared to the placebo group. It was also reported that intrathecal fentanyl had a similar effect compared to sufentanil in terms of the duration of analgesia, and its addition to the local anesthetic resulted in a faster return of motor block and patient consciousness; thus, it seems to be the preferred narcotic drug for CS (6). In a meta-analysis study, Hu et al. found that adding sufentanil to bupivacaine for spinal anesthesia in CS provided a better quality of analgesia than bupivacaine alone (15). Further, other studies have shown that there is no significant difference in the duration of motor block and analgesia between the 2 groups receiving fentanyl and sufentanil in combination with intrathecal bupivacaine (16, 17), which is consistent with the results of the present study. However, the findings of studies conducted by Farzi et al. (6) and Khare and Rupera (18) represented that the duration of sensory and motor block and analgesia was longer in the sufentanil group than in the intrathecal fentanyl group. This discrepancy in results can be explained by a wide range of applied methods, the dose of the administered drugs, and the characteristics of the evaluated patients. However, overall, the results of almost all previous studies support the effectiveness of fentanyl and sufentanil in increasing the duration of analgesia in women undergoing CS.

In a clinical study, Manouchehrian et al. revealed that intrathecal fentanyl had comparable analgesia, quicker onset, and more fulfillment but shorter duration than sufentanil during labor (19). A systematic review reported that sufentanil led to a longer duration of analgesia in spinal and epidural anesthesia compared to fentanyl (20). Ropivacaine blocks conduction in sensory and motor nerves, while sufentanil disrupts pain transmission in the dorsal horn; therefore, adding sufentanil to ropivacaine can synergistically increase the duration of sensory block and analgesia (21). Our results indicated that simultaneous administration of spinal fentanyl and sufentanil, together with local anesthetics, can be used for postoperative analgesia. However, definitive conclusions should be made with caution due to the limited studies in this field. In the present study, BP and HR did not change significantly in the 2 groups at different time intervals, except for the fifth minute when SBP and DBP were lower in the sufentanil group compared to the fentanyl group. In the sufentanil group, SBP and DBP decreased upon administering spinal anesthesia and at the fifth minute, which immediately increased with proper management after 10 minutes. In the fentanyl group, a decrease in SBP and HR was observed only at the fifth minute compared to before the administration of spinal anesthesia. Other studies have also reported similar results, indicating no significant difference between the intrathecal administration of fentanyl and sufentanil in combination with bupivacaine for spinal anesthesia in terms of intraoperative hemodynamic variables (15, 16, 22). The present study indicated that the administration of these drugs resulted in hemodynamic stability during surgery. However, a higher proportion of participants in the sufentanil group (26.9%) experienced side effects compared to the fentanyl group (12%). Notably, the incidence of pruritus was significantly higher in the sufentanil group than in the fentanyl group.

In the study by Miao et al. on pregnant women undergoing elective CS, epidural sufentanil (0.5 μg/mL), along with ropivacaine (0.1% and 0.15%) for postoperative analgesia, caused pruritus in 9.3% of cases (21). Different sample sizes and methods of drug prescribing may explain the differences in results. Likewise, in the study by Cai et al. (23), sufentanil (22.5 μg), along with ropivacaine (0.1%) for labor analgesia, caused pruritus in 3.3% of cases, which is extremely less than the rate obtained in the present study and can explain using a lower dose of sufentanil in the above-mentioned study. Additionally, considering that pruritus can be more unpleasant for the patient than pain, fentanyl is preferable to sufentanil in this regard.

In some other studies, the incidence of pruritus was higher in the sufentanil (5 μg) group than in the intrathecal fentanyl group (25 μg), but other side effects (such as nausea and vomiting) were not significantly different between the 2 groups (14, 15, 24). In addition, in a meta-analysis study, Fonseca et al. showed that respiratory depression was a rare occurrence and easily controllable when fentanyl or sufentanil was added to local anesthetics (0.7%) (14). These results are in line with the current study.

On the other hand, the results of Farzi et al. showed that adding fentanyl or sufentanil to intrathecal bupivacaine in women undergoing CS did not cause severe side effects (6). Pruritus was observed in only 5 cases (16.7%) in the fentanyl group, and no pruritus was reported in the sufentanil and placebo groups. Further, the incidence of nausea, vomiting, and respiratory depression did not differ significantly between the 3 groups (6). In this study, side effects were evaluated within 24 hours after the operation, which could be the reason for the difference in the results of the present study. Furthermore, differences in local anesthetics and patient characteristics, as well as patients’ self-reports regarding the severity of side effects (including pruritus), can cause discrepancies in results. Uppal et al. found that the administration of intrathecal fentanyl in women undergoing CS caused a 6-fold increase in pruritus compared to local anesthetic alone with different doses of bupivacaine (10). While pruritus can be uncomfortable for patients, the analgesic benefits of fentanyl and sufentanil are considered to outweigh any discomfort caused by pruritus or other potential side effects. Moreover, due to the lipophilic nature (solubility in fat) of fentanyl and sufentanil, pruritus presents only temporarily and resolves quickly. In the study by Lord Lasemi et al., although pruritus was reported as a side effect of the intrathecal administration of fentanyl and sufentanil in women undergoing CS, no evidence was found in favor of a higher prevalence of pruritus in the sufentanil group compared to the fentanyl group (25).

Consequently, there is moderate to high-quality evidence supporting the safety and efficacy of adding lipophilic opioids (i.e., fentanyl and sufentanil) to local anesthetics in spinal anesthesia (14). However, studies have used different doses of local anesthetics and opioids (mostly bupivacaine or low bupivacaine and lidocaine), as well as fentanyl and sufentanil. The heterogeneity of methods used in these studies makes it difficult to compare their results. Nevertheless, the overall findings of this study suggest that the combination of drugs used to control pain in patients was effective and safe, with no significant adverse effects. Thus, it can be used as a safe and effective method to increase the duration of post-cesarean delivery analgesia.

The present study has a number of limitations. First, we did not consider factors affecting pain after CS, including the level of anxiety. Second, pain and side effects were evaluated in the short term. Additionally, the incidence of urinary retention was not investigated in this study because the urinary catheter was inserted before the operation and was removed after the patient was able to walk. Finally, this study was conducted in only 1 treatment center with a relatively small number of samples. Future studies are therefore recommended to recruit a larger number of participants and should be conducted in more than 1 center to obtain more accurate results in this regard.

### 5.1. Conclusions

Adding fentanyl or sufentanil to intrathecal ropivacaine could equally increase the duration of analgesia while not causing severe side effects. In addition, adding sufentanil and fentanyl to ropivacaine could keep the patient’s hemodynamic status stable during surgery. However, fentanyl seems to be the preferred drug for increasing the duration of analgesia in CS with spinal anesthesia since it has the same effect as sufentanil in terms of the duration of analgesia and the return of motor block and has fewer hemodynamic fluctuations and side effects (pruritus) compared to sufentanil.

## Data Availability

The data presented in this study are uploaded during submission as a supplementary file and are openly available for readers upon request.

## Clinical Trial Registration Code:

The trial protocol was approved by the Iranian Registry of Clinical Trials (code: IRCT20210827052297N1).
